# Effect of Yeast-Fermented Citrus Pulp as a Protein Source on Nutrient Intake, Digestibility, Nitrogen Balance and In Situ Digestion Kinetics in *Nili Ravi* Buffalo Bulls

**DOI:** 10.3390/ani11061713

**Published:** 2021-06-08

**Authors:** Awais Shabbir, Muhammad Sharif, Khurram Ashfaq, Amjad Islam Aqib, Muhammad Saeed, Alessandro Di Cerbo, Mahmoud Alagawany

**Affiliations:** 1 Institute of Animal and Dairy Sciences, University of Agriculture, Faisalabad 38000, Pakistan; awaismughal4545@gmail.com; 2 Department of Clinical Medicine and Surgery, University of Agriculture, Faisalabad 38000, Pakistan; drkhurramashfaq33@gmail.com; 3 Department of Medicine, Cholistan University of Veterinary and Animal Sciences, Bahawalpur 63100, Pakistan; amjadwaseer@cuvas.edu.pk; 4 Faculty of Animal Production and Technology, Cholistan University of Veterinary and Animal Sciences, Ba-hawalpur 63100, Pakistan; msaeed@cuvas.edu.pk; 5 School of Biosciences and Veterinary Medicine, University of Camerino, 62024 Matelica, Italy; 6 Department of Poultry, Faculty of Agriculture, Zagazig University, Zagazig 44511, Egypt

**Keywords:** single cell protein, kinetics, yeast, citrus pulp

## Abstract

**Simple Summary:**

A study was carried out to evaluate the effect of single cell protein (SCP) supplement as a protein source on nutrient intake, digestibility, nitrogen balance and in situ digestion kinetics in four *Nili Ravi* buffalo bulls. Four iso-caloric and iso-nitrogenous concentrates containing 3, 6, 9 and 12% of *Saccharomyces cerevisiae*-fermented citrus pulp were formulated and provided for 12 weeks. Chemical composition of fermented citrus pulp appeared as an excellent source of protein as no significant difference was observed on dry matter intake, digestibility of nutrients, SCP, ruminal pH and ammonia nitrogen. It is concluded that SCP could be used in the concentrate diet of ruminant up to 12%. Furthermore, the SCP has the potential of an alternative protein source in animal diet formulation.

**Abstract:**

A study was carried out to evaluate the effect of single cell protein (SCP) supplement as a protein source on nutrient intake, digestibility, nitrogen balance and in situ digestion kinetics in four *Nili Ravi* buffalo bulls. Four iso-caloric and iso-nitrogenous concentrates containing 3, 6, 9 and 12% of *Saccharomyces cerevisiae*-fermented citrus pulp were formulated. All animals were fed a ration with a concentrate/forage ratio of 50:50. Diets were provided ad libitum twice a day as a total mixed ration in a 4 × 4 Latin Square Design. Each experimental period lasted 3 weeks while the overall study 12 weeks. The first 2 weeks of each experimental period were used as adaptation period while the third week as collection period. Chemical composition of fermented citrus pulp appeared as an excellent source of protein. No significant difference was observed on dry matter intake, digestibility of nutrients and SCP among all the treatments. Moreover, no significant effect was observed on ruminal pH and ammonia nitrogen at different times. Rate of disappearance and lag time of in situ dry matter digestion kinetics remained nonsignificant regardless of SCP percentage. Based on results of similar nutrients intake, nutrient digestibility, and ruminal parameters it is concluded that SCP could be used in the concentrate diet of ruminant up to 12%. Furthermore, the SCP has the potential of an alternative protein source in animal diet formulation.

## 1. Introduction

Single cell protein supplementation in the diet of small animals and ruminants has been widely acknowledged during the last decade [1,2,3]. As a single cell the *Saccharomyces cerevisiae,* a unicellular fungus belonging to the fungi kingdom, was selected. Although several yeast species are available on the market, *Saccharomyces cerevisiae* is considered as one of the best for culture production due to its growth and metabolic features [4]. 

*Saccharomyces cerevisiae* is a rich source of enzymes, vitamins and other unknown cofactors that increase the activity of microbes in the rumen [5,6]. It also has a good amino acids profile and is endowed with prebiotic activity [7,8,9]. It also has the ability to compensate vitamin and amino acid deficiencies [10]. Live culture of *Saccharomyces cerevisiae* chemically consists of 93% dry matter, 44.5% crude protein, 1.10% ether extract, 3.50% ash, 2.75% crude fiber and 1990 Kcal/kg metabolizable energy [11]. Moreover, it has a high biological value of protein that in turn improves the nutritional value of feed and makes it a valuable alternative to conventional protein sources [12].

*Saccharomyces cerevisiae* has been added in ruminant diet to increase the number of ruminal bacteria and improve the dry matter intake along with digestibility of fiber and crude protein [13]. Supplementation of yeast culture was also shown to stimulate the growth of beneficial microorganisms in the rumen and reduce urinary nitrogen excretion [14,15,16,17]. 

Yeast supplementation also positively affects the feed intake and digestion process in the rumen [18]. Several studies indicate that yeast-fermented products can replace the conventional protein sources e.g., soybean meal, up to 75% in concentrate ration, which improves rumen fermentation and dry matter intake [19,20,21].

Yeast culture reduces the accumulation of lactic acid and oxygen in rumen to improve fiber digestion and starch utilization [15], thus resulting beneficial for rumen fermentation and nutrient digestion [22,23,24].

Yeast-fermented products have the ability to fully replace soybean meal in concentrated mixtures used for ruminants [25], increase the ruminal pH and ruminal fiber digestion rate extent, which ultimately improves the animal performance [26].

Therefore, yeast-fermented products can be used as a nonconventional protein source in a concentrate ration without any negative impact on nutrients intake, digestibility, nitrogen balance and in situ digestion kinetics in ruminants. The present study aimed to investigate the effect of yeast-fermented citrus pulp as a protein source on nutrients intake, digestibility, nitrogen balance and in situ digestion kinetics in cannulated buffalo bulls.

## 2. Materials and Methods

The research study was conducted on four cannulated *Nili Ravi* buffalo bulls at Raja Muhammad Akram Research Center, University of Agriculture, Faisalabad. Operative procedures and animal care were performed in compliance with the national and international regulations. The protocol was examined and approved prior to the beginning of the study by the Veterinary Ethical Review Committee. The recommendations of the ARRIVE guidelines in animal research were also consulted and considered [27].

Four iso-caloric and iso-nitrogenous concentrates containing 3, 6, 9 and 12% of yeast-fermented citrus pulp were formulated and represented as A, B, C, and D following the National Research Council guidelines [28] (Table 1). The yeast-fermented citrus pulp was prepared according the procedure of Sadh et al. [29].

All animals were fed a ration of concentrate/forage ratio of 50:50 and the chemical composition of forage was 95.4 DM, 6.8% Ash, 93.2% OM, 6.64% CP, and 1.4% EE. The concentrate crude protein ratio was 18% while the crude protein of total mix ration was adjusted to 14%. Bulls were fed diets ad libitum twice a day as a total mixed ration in a 4 × 4 Latin Square Design. Each experimental period lasted 3 weeks. The first 2 weeks were used for adaptation while the third one as collection. Overall study lasted 12 weeks.

Feed and feces were recorded daily and nutrient intake was calculated from the collected samples. Digestibility of nutrients was calculated by total collection method. During each collection period, complete urine and feces were collected on each day for nitrogen balance determination. For the first 2 days of each collection period, ruminal samples were collected from four different locations in the rumen at 3, 6, 9 and 12 h post feeding and pH values were determined. Portable pH meter (Orion portable Hanna HI 8314, Hanna industries, Romania model 230A, pH triode electrode; Orion Research, Inc., Boston, MA, USA) was used for immediate ruminal pH determination. Ruminal samples were squeezed through four layers of cheesecloth and 50 mL of the liquid were acidified with 3 mL of 6 N HCl to terminate fermentation. Samples were then used to determine ruminal ammonia by Kjeldhal’s method [30].

In situ experiment was conducted to determine the digestion kinetics of yeast-fermented product using ruminally cannulated buffalo bulls. During this experiment, 10 × 23 cm nylon bags, with an average pore size of 50 μm, were used to determine dry matter (DM) and neutral detergent fiber (NDF) disappearance rate and extent. For each time point, 5 g of yeast-fermented product sample were weighed into bags, in triplicate. Two bags were used to determine DM and NDF disappearance while one bag served as blank. The bags were closed and tied with braided nylon fishing line. To remove soluble or 50-μm filterable materials, the bags were dipped into a specific amount of tap water for 15 min just before ruminal incubation. Weight loss due to dipping was expressed as pre ruminal dry matter disappearance. Three bags for each diet were incubated in the rumen of buffalo bulls for 0, 1, 2, 4, 6, 10, 16, 24, 36, 48 and 96 h intervals in reverse order and removed all at the same time. After rumen removal, bags were washed with running tap water until the rinse was clear. The bags were dried and residues were transferred to 100 mL cups and stored until the analysis. In situ digestion kinetics parameters, e.g., rate, lag and extent of DM and neutral detergent fiber NDF disappearance, were calculated for each period individually.

The proximate composition of yeast-fermented citrus pulp was determined according to AOAC [30]. The collected feed samples and rumen residues were analyzed for DM and NDF. For DM and ash determination, hot air oven was used at 105 °C for 24 h and at 600 °C for 3 h, respectively. The nitrogen content was determined by Kjeldhal’s method [30] and CP was calculated as N × 6.25. The NDF was determined by the procedure described by Van Soest et al. with sodium sulphite [31]. 

### Statistical Analysis

Data were subjected to one-way analysis of variance using Latin Square Design and treatment means were compared by using Tukey’s multiple comparisons test. A *p* < 0.05 was considered significant.

## 3. Results

### 3.1. Nutritional Composition

Nutritional values for fermented citrus pulp are given in Table 2 on dry matter basis. Results revealed that fermented citrus pulp had an excellent nutritional profile, resulting in a good source of protein, either extract and energy. 

### 3.2. Nutrient Intake and Nutrient Digestibility

Results indicated no significant difference on dry matter intake among all the treatments. However, the highest numerical values for dry matter intake (DMI) were observed for diet D followed by diets C, B and A, respectively (Table 3). Use of yeast-fermented citrus pulp as a protein source in *Nili Ravi* buffalo bull’s diets did not affect crude protein intake. Neutral detergent fiber and acid detergent fiber (ADF) intakes remained unaltered among all dietary treatments. Dry matter digestibility showed no significant difference due to different levels of yeast-fermented citrus pulp as a protein source. Digestibility of CP, NDF and ADF also remained nonsignificant among all the treatments.

### 3.3. Nitrogen Balance

Inclusion of SCP in buffalo bull’s diets showed no significant difference among all dietary treatments (Table 4). Similarly, nitrogen in feces and urine remained unaffected among different treatments. Further, no significant effect was observed on nitrogen retention at different levels of yeast-fermented citrus pulp, despite the higher values observed in the diets.

### 3.4. Ruminal pH and Ammonia Nitrogen

No significant effect was observed on ruminal pH and ammonia nitrogen at 3 h postprandial and at all levels of yeast-fermented citrus pulp in buffalo bulls (Table 5). A similar trend was also observed on ruminal characteristics at 6 and 9 h post feeding among all dietary treatments.

### 3.5. In Situ Digestion Kinetics

Rate of disappearance and lag time of in situ dry matter digestion kinetics remained nonsignificant in buffalo bulls fed different levels of SCP supplement. In situ dry matter digestion kinetics extent also remained unaffected among the dietary treatments. Similarly, rate of disappearance and lag time of in situ NDF digestion kinetics remained nonsignificant among all dietary treatments. As for dry matter, no significant effect was observed on digestion extent of in situ NDF digestibility (Table 6).

## 4. Discussion

Citrus pulp is a poor source of crude protein; however, fermentation can improve its value [32,33,34]. The increase in protein content after fermentation was presumably due to extracellular protein secretion, constituents metabolization or multiplication in the form of SCP by *Saccharomyces cerevisiae* [35]. Furthermore, the increase in growth and proliferation of the microorganisms in the fermenting substrates might possibly account for the apparent increase in the protein content of the fermented peels [36]. These results correlate with the findings of Oboh and Akindahunsi who observed increase in protein level in cassava products [37]. This could be due to possible secretion of some extracellular enzymes (proteins) such as amylase and cellulase into the substrates, which break the starch and other polysaccharides into simpler sugars that are easily metabolized by yeast as a carbon source. 

As far as concerns the nutrient intake, results of the current study are in accordance with the findings of Wanapat et al. who observed nonsignificant effects on dry matter intake after replacement of soybean meal with yeast-fermented cassava chips concluding that soybean meal could be fully replaced by yeast-fermented cassava chips without any adverse effect [38]. Similarly, Boonnop et al. reported that yeast-fermented cassava pulp could fully replace soybean meal without any negative effect on nutrient intake [25]. Additionally, Gobindram et al. investigated the effect of dried citrus pulp on the diet of lambs and concluded that dried citrus pulp had no significant effect on dry matter intake [39]. However, Williams et al. found that supplementation of yeast (10 g/d) in the diet of dairy cows increased dry matter intake [6]. Similarly, in dairy cows, Putnam et al. reported that supplementation of yeast culture (10 g/d) increased dry matter intake as compared to control group [40]. Pinos-Rodriguez et al. observed that supplementation of *Saccharomyces cerevisiae* increased DMI in ruminants [41]. Crosswhite et al. found that DM intake was higher in animals fed a diet supplemented with dried citrus pulp [42]. The reasons for increased intake might be likeness of animals for yeast-fermented citrus pulp due to its specific smell and taste as well as the better palatability of citrus pulp [43].

Results concerning nutrient digestibility are in close resemblance with Khampa et al. who observed that animals fed yeast-fermented cassava chips had no significant effect on nutrient digestibility [20]. Similarly, Wanapat et al. found that addition of yeast-fermented cassava chips in the diet of animals had no significant effect on DM and NDF digestibility [38]. Studies on yeast-fermented cassava pulp substitution for soybean meal in the diet of ruminants also indicated no significant effect on DM, NDF and ADF digestibility [25]. Animals fed dried citrus pulp had no significant effect on nutrient digestibility [44]. De Lima et al. found that supplementation of dry yeast had no significant effect on DM, NDF and ADF digestibility [45]. These studies indicated that microorganism and substrate alone also have no adverse effect on animals’ performance. 

Conversely, Ghazanfar et al. reported that addition of *Saccharomyces cerevisiae* alone in the ration improved the digestibility of DM, CP, CF, NDF and ADF as compared to control group [46]. Ullah et al. also reported positive influence of *Saccharomyces cerevisiae* on nutrient digestibility [47]. The increased digestibility could be attributed to the increased nitrogen content of the rumen, which improved the growth of microbial population and led to increase in digestibility. In addition, Haddad and Goussous (2005) observed that supplementation of yeast culture improved CP and NDF digestibility compared to control group [48]. This can be ascribed to the increased concentration (5–40 times) of cellulolytic microorganisms in the rumen of yeast-supplemented animals rather than the nonsupplemented ones, resulting in a higher nutrient digestibility [14]. 

Our results regarding rumen characteristics in buffalo calves are in agreement with Wanapat et al. who reported that the addition of yeast-fermented cassava chips in the diet of animals had no significant effect on rumen pH [38]. Similarly, Khampa et al. also found that animals fed yeast-fermented cassava chips had no significant effect on ruminal pH and ammonia when compared with other conventional expensive protein sources (rice straw and rice bran) [19]. Other studies on crossbred native cattle, also observed that supplementation of yeast-malate-fermented cassava pulp and cassava as well as yeast-fermented lemon pulp did not induce any significant difference in the rumen pH regardless of treatments [20,49]. Conversely, Dolezal et al. found higher ruminal pH when yeast culture was added in the diet of dairy cows [5]. Boonnop et al. reported that yeast-fermented cassava pulp substitution for soybean meal in the diet of ruminants significantly increased ruminal ammonia regardless of treatments [25]. 

Dealing with nitrogen balance, our results are in line with other authors who observed that yeast itself and yeast-fermented product had no significant effect on nitrogen balance [50,51]. This might be due to the nonsignificant effect of yeast on nutrient intake and digestibility. However, Sawsan et al. found that addition of yeast culture in the lambs’ ration had more nitrogen balance as compared to control group [52]. Lambs had higher nitrogen balances raised on ration supplemented with yeast culture [53]. The higher nitrogen balance may be due to higher production of microbial protein synthesis as a result of yeast culture [54]. 

In situ digestion kinetics results revealed that SCP supplement had no significant effect on rate of disappearance, lag time and extent of digestion. Results are in the line with the findings of Lehloenya et al. who reported that supplementation of yeast culture in the diet of steers had no significant effect on ruminal digestion kinetics [51]. Doreau and Jounay found that addition of yeast in the diet increased ruminal dry matter content [55]. However, DM and NDF degradability was not significantly improved. Similarly, Olson et al. observed that supplementation of yeast culture did not affect rate or lag time of NDF disappearance [56]. Corona et al. recorded that animals fed yeast culture with basal diet (sorghum grain and corn stovers) had no significant on DM and NDF degradability [57].

## 5. Conclusions

Based on our results about similar nutrients intake, nutrient digestibility, and ruminal parameters it is concluded that yeast-fermented citrus pulp could be used in the concentrate diet of ruminants. Our results indicated that yeast-fermented citrus pulp can be used successfully up to 12% of the concentrate in the diet of bulls without any adverse effect on growth performance and feed intake. Thus, the yeast-fermented citrus pulp holds the potential of an alternative protein source and economic ingredient in animals’ diet.

## Figures and Tables

**Table 1:** Experimental diet and chemical composition.

Ingredients %	Concentrate Diets ^1^			
	A	B	C	D
Corn	9.8	8.8	9.9	9
Cotton seed cake	1	1	1	1
Maize oil cake	1	1	1	1
Corn gluten 30%	0.5	0.5	2	0.5
Wheat bran	9	7.6	4	3
Canola meal	30	30	30	30
Yeast-fermented citrus pulp	3	6	9	12
Rice polish	25	25	25	25
Sunflower meal	0.5	0.5	0.5	0.5
Molasses	15	15	14	15
Oil	1.6	1.2	0.5	0
Urea	0.6	0.4	0.1	0
Premix	3	3	3	3
Total weight	100	100	100	100
Chemical composition, %				
Crude protein	18.38	18.41	18.37	18.40
ME Kcal/kg	2869	2862	2859	2840
Neutral detergent fiber	23.92	23.81	23.50	23.05
Acid detergent fiber	13.14	13.21	13.16	13.14
Ash	9.7	9.75	9.81	9.58

^1^ A, B, C and D represent 3%, 6%, 9% and 12% inclusion of yeast-fermented citrus pulp, respectively.

**Table 2:** Composition of yeast-fermented citrus pulp.

Item	Composition
Dry matter	920 g/kg
Crude protein	305 g/kg
Metabolizable energy	3040 Kcal/kg
Ether extract	47 g/kg
Neutral detergent fiber	201.5 g/kg
Acid detergent fiber	106 g/kg
Ash	67.2 g/kg

**Table 3:** Effect of different levels of yeast-fermented citrus pulp on nutrient intake and digestibility.

Items	Concentrate Diets ^1^				SEM	*p*-Value
	A	B	C	D		
Intake (kg/day)						
Dry matter	12.96	13.36	13.56	13.99	0.75	0.97
Crude protein	2.41	2.46	2.5	2.6	0.14	0.98
Neutral detergent fiber	7.13	7.38	7.5	7.67	0.44	0.98
Acid detergent fiber	3.42	3.52	3.57	3.72	0.21	0.97
Digestibility (kg/day)						
Dry matter	67.86	69.57	69.30	69.13	0.52	0.70
Crude protein	68.92	69.16	70.53	68.63	0.33	0.17
Neutral detergent fiber	60.03	57.43	56.29	56.45	1.16	0.71
Acid detergent fiber	50.83	49.14	48.42	48.53	1.98	0.87

^1^ A, B, C and D represent 3%, 6%, 9% and 12% inclusion of yeast-fermented citrus pulp, respectively.

**Table 4:** Effect of different levels of yeast-fermented citrus pulp on nitrogen balance in cannulated buffalo bulls.

Parameters (g/Day)	Concentrate Diets ^1^				SEM	*p*-Value
	A	B	C	D		
Nitrogen intake	454.57	463.02	438.10	460.40	29.30	0.99
Nitrogen in feces	73.48	74.30	60.52	65.27	5.64	0.85
Nitrogen in urine	189.03	222.97	205.20	213.67	13.32	0.83
Nitrogen retention	165.75	172.37	172.37	181.45	16.39	0.95

^1^ A, B, C and D represent 3%, 6%, 9% and 12% inclusion of yeast-fermented citrus pulp, respectively.

**Table 5:** Effect of different levels of yeast-fermented citrus pulp on ruminal pH and ammonia nitrogen in cannulated buffalo bulls.

Ruminal pH	Concentrate Diets ^1^				SEM	*p*-Value
	A	B	C	D		
3 h	7.32	7.42	7.46	7.52	0.06	0.74
6 h	7.42	7.25	7.27	7.32	0.07	0.86
9 h	7.15	7.05	7.01	7.03	0.05	0.82
12 h	7.58	7.36	7.43	7.18	0.07	0.23
Ammonia (mg/dL)						
3 h	39.10	47.17	43.35	41.80	4.94	0.46
6 h	58.65	42.92	52.70	48.87	4.79	0.09
9 h	48.87	48.02	45.47	47.17	4.68	0.83
12 h	42.92	43.77	51.0	39.95	3.21	0.14

^1^ A, B, C and D represent 3%, 6%, 9% and 12% inclusion of yeast-fermented citrus pulp, respectively.

**Table 6:** Effect of different levels of yeast-fermented citrus pulp on in situ nutrient digestibility in cannulated buffalo bulls.

Item	Concentrate Diets ^1^				SEM	*p*-Value
	A	B	C	D		
Dry matter digestibility						
Rate of disappearance (%h)	6.07	10.55	8.62	10.82	0.70	0.05
Lag time (h)	14.99	8.12	11.68	9.17	1.01	0.06
Digestion extent (%)	94.77	93.04	94.37	92.57	0.63	0.64
Neutral detergent fiber digestibility						
Rate of disappearance (%h)	7.90	8.85	8.30	7.72	0.26	0.46
Lag time (h)	4.94	3.47	3.38	4.51	0.36	0.44
Digestion extent (%)	90.93	90.41	91.75	89.81	0.47	0.53

^1^ A, B, C and D represent 3%, 6%, 9% and 12% inclusion of yeast-fermented citrus pulp, respectively.

## Data Availability

The data presented in this study are available on request from the corresponding authors.
