# Correction: Shabbir et al. Effect of Yeast-Fermented Citrus Pulp as a Protein Source on Nutrient Intake, Digestibility, Nitrogen Balance and In Situ Digestion Kinetics in Nili Ravi Buffalo Bulls. *Animals* 2021, *11*, 1713

**DOI:** 10.3390/ani12192514

**Published:** 2022-09-21

**Authors:** Awais Shabbir, Muhammad Sharif, Khurram Ashfaq, Amjad Islam Aqib, Muhammad Saeed, Alessandro Di Cerbo, Mahmoud Alagawany

**Affiliations:** 1 Institute of Animal and Dairy Sciences, University of Agriculture, Faisalabad 38000, Pakistan; 2 Department of Clinical Medicine and Surgery, University of Agriculture, Faisalabad 38000, Pakistan; 3 Department of Medicine, Cholistan University of Veterinary and Animal Sciences, Bahawalpur 63100, Pakistan; 4 Faculty of Animal Production and Technology, Cholistan University of Veterinary and Animal Sciences, Ba-hawalpur 63100, Pakistan; 5 School of Biosciences and Veterinary Medicine, University of Camerino, 62024 Matelica, Italy; 6 Department of Poultry, Faculty of Agriculture, Zagazig University, Zagazig 44511, Egypt

## Correction of Author’s Name

Awais Shabbir was mistakenly written as “Muhammad Awais” in the original publication [1]. The corrected Author Contributions Statement appears here.

**Author Contributions:** Conceptualization, A.S., M.S. (Muhammad Sharif), K.A., A.I.A. and M.S. (Muhammad Saeed); methodology, A.S., M.S. (Muhammad Saeed), K.A., A.I.A. and M.S. (Muhammad Sharif); software, A.S. and M.S. (Muhammad Saeed); validation, A.S., M.S. (Muhammad Sharif), K.A., A.I.A. and M.S. (Muhammad Saeed); investigation, A.S., M.S. (Muhammad Sharif), K.A., A.I.A. and M.S. (Muhammad Saeed); resources, A.D.C. and M.A.; data curation, A.S., M.S. (Muhammad Sharif), K.A., A.I.A. and M.S. (Muhammad Saeed); writing—original draft preparation, A.S., M.S. (Muhammad Sharif), K.A., A.I.A. and M.S. (Muhammad Saeed); writing—review and editing, A.D.C. and M.A.; visualization, A.D.C. and M.A.; supervision, A.D.C. and M.A.; project administration, A.D.C. and A.S. All authors have read and agreed to the published version of the manuscript.

## Text Correction

There was an error in the original publication. The name of pH meter “Orion portable pH meter model 230A, pH triode electrode; Orion Research, Inc., Boston, MA, USA” was mistakenly written.

A correction has been made to the above-mentioned error in Materials and Methods, *paragraph number 4 and line numbers 6–8:*

“Portable pH meter (Orion portable Hanna HI 8314, Hanna industries, Romania model 230A, pH triode electrode; Orion Research, Inc., Boston, MA, USA) was used for immediate ruminal pH determination”

The authors apologize for any inconvenience caused and state that the scientific conclusions are unaffected. This correction was approved by the Academic Editor. The original publication has also been updated.
